# Smoking History Decreases Survival in Patients with Squamous Cell Carcinoma of the Mouth: A Retrospective Study with 15 Years of Follow-up

**DOI:** 10.31557/APJCP.2019.20.6.1781

**Published:** 2019

**Authors:** Naiara Colares, Déborah Franklin Souza Rodrigues, Milena Oliveira Freitas, Thinali Sousa Dantas, Maria do Perpétuo Socorro Saldanha Cunha, Fabrício Bitu Sousa, Paulo Goberlânio de Barros Silva

**Affiliations:** 1 *Department of Dentistry, Unichristus, Rua João Adolfo Gurgel 133, *; 2 *Haroldo Juaçaba Hospital, Ceará Cancer Institute, Rua Papi Júnior, 1222, Rodolfo Teófilo, Fortaleza, Ceará, Brazil. *

**Keywords:** Survival analysis, survival rate, oral neoplasms, squamous cell carcinoma, smoking

## Abstract

**Background:**

The purpose of this study was to evaluate the influence of smoking history on the clinical-pathological, sociodemographic and prognostic characteristics of patients with oral squamous cell carcinoma (SCC).

**Materials and Methods:**

A retrospective cohort study was carried out with the records of 136 smokers with SCC and 68 nonsmokers with oral SCC who were diagnosed and treated at Haroldo Juaçaba Hospital (2000-2014). Data on patient sex, age, race, education level, tumor location, tumor size, lymph node involvement, distant metastasis, treatment type, marital status, method of health care access (public or private health systems) and overall survival (15 years) were analyzed by the X² test, Mantel-Cox tests and multinomial and Cox logistic regression models (SPSS 20.0, p <0.05).

**Results:**

Smoking history was directly associated with male sex (p <0.001), low levels of education (p = 0.001), tumors of the mouth and palate (p = 0.001), stage T3/4 tumors (p = 0.014), lymph node metastasis (N+) (p = 0.024), palliative treatment (p = 0.024) and receiving health care through the public health system (p = 0.006), with education level being the only independently associated factor (p = 0.039). Lower survival was observed in patients who were smokers (p = 0,002), with low levels of education (p = 0.001), who had stage T3/4 tumors (p = 0.004), with N+ (p = 0.021), and had received palliative treatment (p = 0.002). Age (>65 years old, p = 0.015) and T staging (T3/4, p = 0.033) decreased the survival of SCC patients regardless of the other factors.

**Conclusions:**

Smoking history had an independent association with low education level and a history of alcoholism, and survival was negatively associated with older age and larger tumor size, which were more prevalent in smokers.

## Introduction

Cancer is a global public health issue, causing approximately 8.8 million deaths per year. According to the World Health Organization (WHO), the countries that suffer most from the disease are low-income and middle-income countries. Mouth cancer is among the 10 most frequent cancers in the world population (Stewart and Wild, 2014), and its incidence is increasing. In 2018, 14,700 new cases are expected in Brazil, including 11,200 men and 3,500 women. High incidence rates are related to a number of risk factors, such as age, sex, alcoholism and smoking (Vargas-Ferreira et al., 2012; Ferreira et al., 2012). Smoking is one of the main factors that has contributed to the development of squamous cell carcinoma (SCC), which is the main histological type of cancer observed in the oral cavity (Al-Jaber et al., 2016).

Tobacco consumption, chewed or smoked, is recognized as the main risk factor for the onset of oral SCC (Warnakulasuriya et al., 2005) and is associated with a number of cytotoxic and genotoxic effects (Castagnola et al., 2017; Motgi et al., 2014). Tobacco smoke, in particular, contains carcinogens that bind to DNA, generating DNA adducts (Phillips and Venitt, 2012), which are mutations that are capable of activating cellular oncogenes or inactivating tumor suppressor genes (Hecht, 2008; Chen et al., 2013). Moreover, in human genes mutated by smoking, the regulation of micro (mi) RNAs may be affected, with changes in the domains that control all cellular, physiological and developmental processes (Wienholds; Plasterk, 2005), being established as the main regulators of the oncogenic potential in cells. Changes in genetic and epigenetic levels in the complex enzymatic mechanism involved in the biogenesis of miRNAs result in aberrant expression in patients who smoke (Castagnola et al., 2017).

Recent studies of smokers show that life expectancy in this group is seven years lower than that of nonsmokers (Streppel et al., 2007). Smoking cessation promotes a significant reduction in the mortality rate before the age of 35 and on a smaller scale in the age group over 65 years old, in addition to producing numerous health benefits; thus smoking cessation is a cost-effective intervention (Sales et al., 2006).

The act of smoking involves a series of modifications in the sociodemographic and educational profile. The prevalence and type of tobacco use among men and women varies in countries and in socioeconomic subgroups, with the prevalence being lower in women than in men in all countries (Doku, Darteh and Kumi-Kyereme, 2013). Additionally, smoking and smokeless tobacco use are associated with older age, lower education levels and poverty (Sreeramareddy et al., 2014).

Educational status and sociodemographic profile modify a patient’s smoking history, interfering with the prognosis of patients with oral cancer. Thus, the present study aims to associate smoking history as an influencing agent in the survival of patients with oral cancer treated at a regional reference center in northeast Brazil.

## Materials and Methods

*Study design*

This study is characterized as a quantitative, observational, analytical, retrospective cohort study. The data were extracted through the analysis of medical records of 204 patients with oral SCC who were patients at the Haroldo Juaçaba Hospital (Cancer Institute of Ceará) from 2000 to 2014 (15 years of follow-up). From this sample (used for convenience), sociodemographic data such as age, sex, race, education, marital status, smoking status, and method of hospital admission were analyzed. Available clinical data were also collected from the medical records, including oral cavity location, tumor clinical staging (TNM), overall survival and treatment performed, which is divided into surgery, surgery associated with radiotherapy, radiotherapy, radiotherapy associated with chemotherapy, surgery associated with radiotherapy and with chemotherapy or no treatment.

The location of the primary tumor was classified as recommended by the WHO, according to the international classification of diseases, which were the lip, gingiva, anterior or posterior thirds of the tongue, hard palate, floor of the mouth and other. The TNM was defined according to a proposal of the National Cancer Institute that determines TNM, with T being the tumor size, N assessing the lymph node involvement and M related to distant metastases.

Survival was calculated as the time from the treatment start date (day, month and year) to the date of death (day, month and year) in number of weeks for statistical analysis (Dantas et al., 2016).

*Statistical analysis*

The data were analyzed using the Statistical Packing for Social Sciences (SPSS) software, version 20.0 for Windows, adopting a confidence interval of 95% and using Fisher’s exact test, Pearson’s chi-square test, and the Mantel-Cox log-rank test for bivariate analysis, as well as multinomial logistic regression models and Cox’s Regression for multivariate analysis.

*Ethical Correlations*

The project was submitted to and approved by the Ethics Committee at the Cancer Institute of Ceará, filed under the number 70596317.6.0000.5528 according to the resolutions of law 466/12 that regulates research with humans.

## Results

*Characterization of the sample of smokers and nonsmokers with oral SCC*

Within the assessed period, there were 979 records of patients with oral SCC. Among these, 204 had information on smoking history (present or absent), with a total of 136 patients who had a history of smoking and 68 patients with no smoking history (smoking:nonsmoking ratio of 2:1). Most of the patients were male, and males patients had a higher proportion of having a smoking history (n=98, 71.1%, p = 0.001).

Most patients were up to 65-year-old, with no difference in age distribution between the groups (p = 0.368). The most frequent tumor site in the nonsmokers was the posterior 2/3 of the tongue (50.0%), and in the smokers, it was the floor of the mouth (39.0%) and the palate (13.2%) (p = 0.001).

The racial distribution of the sample did not show a significant difference between the two groups, with the majority of patients having brown skin (p = 0.124). The degree of education showed that a significant majority of nonsmokers had a high school diploma (n=17, 25.0%) and a high percentage of smokers who were illiterate (n=37, 27.2%) (p = 0.001).

The marital status did not present a statistically significant difference between the two groups (p = 0.072). Nonsmokers were given greater access to health care through private plans (n=51, 52.0%) (p = 0.006).

Clinically, in nonsmoking patients, most tumors were diagnosed as T1-T2 sizes (56.0%) (p = 0.014), without lymph node metastasis (n=27, 55.1%) (p = 0.024), and a low prevalence of distant metastasis was observed in both groups (p = 1.000).

Regarding the type of treatment, nonsmoking patients complied with no treatment, surgery or surgery plus radiotherapy (RT), with RT associated with chemotherapy (CT) being the most common treatment (n=22, 32.4%). In smoking patients, the treatments that were most significantly used were RT and RT associated with CT (p = 0.024).

Alcohol use history was significantly more prevalent in smokers (n=62, 91.2%) (p<0.001), whereas the majority of nonsmokers had no habit of drinking alcohol (n=97, 71.3%).

In a multivariate analysis, the factors that showed a significant association with smoking regardless of the other factors were education level (illiterate, p = 0.039) and alcohol use history (p = 0.001) (Table 3).

*Factors modifying survival over 15 years of oral SCC*

Survival after 15 years was 59.8%, with an average time of 83.90 ± 6.29 months. Having a history of smoking significantly reduced this rate (p = 0.002): in smokers, the mean survival time was 67.01 ± 7.00 months, and in nonsmokers, the survival time was 103.55 ± 8.94 months.

Sex (p = 0.085), age (p = 0.087), race (p = 0.112) and tumor location (p = 0.066) did not influence the survival rate. Educational level had a direct relationship with survival time (p = 0.001), with illiterate patients having the shortest survival time (41.01 ± 7.23 months), followed by patients with an elementary school education (88.85 ± 8.04 months) and patients who completed high school having the highest survival mean time (99.43 ± 11.58 months).

Regarding the TNM classification, patients with T3 and T4 tumors had a shorter survival time of 70.80 ± 8.69 months compared to those with T1 and T2 tumors who had a survival time of 92.88 ± 8.93 months (p = 0.004). In relation to lymph node metastasis, the presence of some affected lymph nodes reduced the mean overall survival time (54.17 ± 5.81) compared to patients without lymph node metastasis (96.04±10.24 months) (p = 0.021). The presence of distant metastasis did not significantly influence the decrease in survival (p = 0.415).

Concerning the therapeutic modalities, patients who received only RT had a shorter survival time of 34.44 ± 4.20 months (p = 0.002). Marital status was not a significant variable for the reduction in survival time (p = 0.833) nor was the form of entry into the hospital (private or public health system) (p = 0.201). In addition, alcohol decreased the survival time of patients with oral cancer (68.45 ± 7.66 months) (p = 0.021) compared to nondrinkers who had a survival time of 94.07 ± 8.13 months.

In the multivariate analysis, the independent risk factors that delayed overall survival were age over 65 years of age (p = 0.015) and T3/T4 staging (p = 0.033).

**Figure 1:**
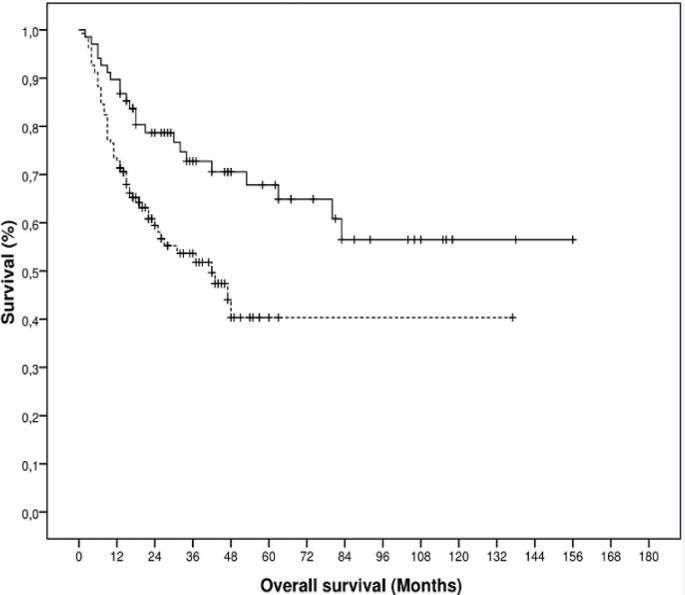
Overall 15-years Survival of Oral SCC Patients in Hospital Haroldo Juaçaca Categorized by Prior Smoking History (Cancer Ceará Institute, 2000-2014). *p, 0.002; Kaplan-Meier Method; Long-Rank Mantel-Cox Test. Dashed line, patients with smoking history; Non-dashed line, patients without smoking history

**Table 1:** Clinic Epidemiological Characterization of Oral SCC Patients with and Without Smoking History in Hospital Haroldo Juaçaca (Cancer Ceará Institute, 2000-2014)

	Smoking				
	No		Yes		p-Value
Sex					
Female	36*	52.9%	38	27.9%	<0.001
Male	32	47.1%	98*	72.1%	
Age					
Up to 65 years old	42	61.8%	75	55.1%	0.368
Above 65 years old	26	38.2%	61	44.9%	
Race					
Brown skin	38	55.9%	91	66.9%	0.124
Not Brown skin	30	44.1%	45	33.1%	
Education					
Illiterate	6	8.8%	37*	27.2%	0.001
Elementary school	45	66.2%	84	61.8%	
High School	17*	25.0%	15	11.0%	
Location					
2/3 tongue anterior	34*	50.0%	29	21.3%	0.001
Floor of the mouth	17	25.0%	53*	39.0%	
1/3 tongue posterior	11	16.2%	24	17.6%	
Palate	3	4.4%	18*	13.2%	
Others	3	4.4%	12	8.8%	
T					
T1-T2	28*	56.0%	35	35.0%	0.014
T3-T4	22	44.0%	65*	65.0%	
N					
N0	27*	55.1%	36	35.6%	0.024
N+	22	44.9%	65*	64.4%	
M					
M0	43	100.0%	65	97.0%	0.519
M1	0	0.0%	2	3.0%	
Treatment					
None	7*	10.3%	13	9.6%	0.024
Surgery	8*	11.8%	3	2.2%	
Surgery + RT	21*	30.9%	32	23.5%	
RT	10	14.7%	31*	22.8%	
RT + CT	22	32.4%	57*	41.9%	
Marital status					
No	51	75.0%	116	85.3%	0.072
Yes	17	25.0%	20	14.7%	
Alcohol use history					
Alcoholic	6	8.8%	97*	71.3%	<0.001
Non- alcoholic	62*	91.2%	39	28.7%	
Entry in the Hospital					
Public health system	26	38.2%	80*	58.8%	0.006
Private plans	42*	61.8%	56	41.2%	

**Table 2:** Influence of Clinic Epidemiological Factors in 15-years Overall Survival of Oral SCC Patients with and Without Smoking History in Hospital Haroldo Juaçaca (Cancer Ceará Institute, 2000-2014)

	Survival in 15 years			
	Time (Months)	n	%	p-Value
Overall survival	83.90±6.29	122	59.8%	-
Smoking				
No	103.55±8.94	46	67.6%	0.002
Yes	67.01±7.00*	76	55.9%	
Sex				
Female	101.13±9.55	50	67.6%	0.085
Male	66.37±6.64	72	55.4%	
Age				
Up to 65 years old	81.89±6.70	75	64.1%	0.087
Above 65 years old	75.86±9.47	47	54.0%	
Race				
Brown skin	59.44±5.51	71	55.0%	0.112
Not Brown skin	100.67±9.38	51	68.0%	
Education				
Illiterate	41.01±7.23*	15	34.9%	0.001
Elementary school	88.85±8.04	83	64.3%	
High School	99.43±11.58	24	75.0%	
Location				
2/3 tongue anterior	98.36±8.33	46	73.0%	0.066
Floor of the mouth	67.46±10.58	37	52.9%	
1/3 tongue posterior	67.47±11.60	23	65.7%	
Palate	42.43±7.81	9	42.9%	
Others	52.36±12.43	7	46.7%	
T				
T1-T2	92.88±8.93	45	71.4%	0.004
T3-T4	70.80±8.69*	43	49.4%	
N				
N0	96.04±10.24	42	66.7%	0.021
N+	54.17±5.81*	45	51.7%	
M				
M0	82.57±7.16	66	61.1%	0.415
M1	81.75±7.12	2	100.0%	
Treatment				
None	51.74±14.39	11	55.0%	0.002
Surgery	105.33±10.34	10	90.9%	
Surgery + RT	105.35±11.20	39	73.6%	
RT	34.44±4.20*	20	48.8%	
RT + CT	71.06±7.93	42	53.2%	
Marital status				
No	84.04±7.23	100	59.9%	0.833
Yes	77.57±11.37	22	59.5%	
Alcoholism				
Alcoholic	68.45±7.66*	57	55.3%	0.021
Non- alcoholic	94.07±8.13	65	64.4%	
Entry				
Public Health System	74.27±6.01	71	67.0%	0.201
Private Plans	73.96±8.55	51	52.0%	

*p<0,05, Mantel-Cox Test (mean±EPM); CT , chemotherapy; RT, radiotherapy.

**Table 3:** Multivariate Analysis of Clinic Epidemiological Factors in Smoking History of Oral SCC Patients with and without Smoking History in Hospital Haroldo Juaçaca (Cancer Ceará Institute, 2000-2014).

	p-Value	Adjusted HR	CI 95%	
Smoking history				
Sex (M)	0.178	-	-	-
Age (>65)	0.819	-	-	-
Race (Not Brown skin)	0.140	-	-	-
Education (Illiterate)	0.039	8.21	1.11	60.63
Location (Tongue)	0.156	-	-	-
T (3/4)	0.397	-	-	-
N (N+)	0.605	-	-	-
M (M+)	1.000	-	-	-
Treatment (Surgery)	0.745	-	-	-
Matrimonial status (Yes)	0.271	-	-	-
Alcoholism (Yes)	<0.001	23.52	6.58	84.07
Entry (Not SUS)	0.119	-	-	-

*p<0,05, multinomial logistic regression; HR, Hazard Risk; CI, Confidence Interval.

**Table 4:** Multivariate Analysis of Clinic Epidemiological and Smoking History in Overall 15-years Survival of Oral SCC Patients in Hospital Haroldo Juaçaca (Cancer Ceará Institute, 2000-2014). Months

	p-Value	HR	CI 95%	
Smoking (Yes)	0.970	-	-	-
Sex (M)	0.657	-	-	-
Age (>65)	*0.015	2.55	1.20	5.43
Race (Not Brown skin)	0.169	-	-	-
Education (Illiterate)	0.078	-	-	-
Location (Tongue)	0.803	-	-	-
T (3/4)	*0.033	2.23	1.07	4.65
N (N+)	0.106	-	-	-
M (M+)	0.973	-	-	-
Treatment (Surgery)	0.821	-	-	-
Marital status (Yes)	0.380	-	-	-
Alcoholism (Yes)	0.100	-	-	-
Entry (Private plans)	0.185	-	-	-

*p<0,05, COX’s Regression Model. HR, Hazard Risk. CI, Confidence Interval.

## Discussion

Oral SCC is the most prevalent malignant neoplasm in this anatomical site (Simard et al., 2014). Our findings corroborate those already existing in the literature that show a higher prevalence of oral SCC in males and a direct association with tobacco use. An epidemiological evaluation of patients with head and neck cancer by Alvarenga et al., (2008) showed that 86% are male and 14% are female, which according to Sales et al., (2006), may be because women are less likely to smoke than men are.

Concerning the age group, the majority of patients in the cohort were up to 65 years of age, which is similar to the demographic of patients in previous studies, showing that the typical oral cancer patients are predominantly men around the fifth and sixth decades of life (Mendez et al., 2012; Santos et al., 2010; Tandon et al., 2017). Furthermore, there is also an association seen in this study that directly relates tobacco history and alcohol intake (Durazzo et al., 2005). In our study, a significant association between smoking and alcohol use was also observed, which is directly associated with the transformation of potentially malignant lesions into malignant ones, acting as a promoting agent for the development of oral cancer (Dobrossy, 2005; Shiu et al., 2004).

The primary sites of tumors with the highest prevalence were the floor of the mouth, the tongue and the palate. In the study by Dedivitis et al., (2004), the primary site of oral tumors was analyzed, and the tongue and floor of the mouth were the most affected. According to Andrade et al., (2015), the anatomical sites most affected by SCC of the mouth were the tongue, followed by the floor of the mouth and the lower lip. Ha and Califano (2004) also showed in their study that the most common locations for oral squamous cell carcinoma were the anterior third of the tongue, followed by the lips, the floor of the mouth, and the hard palate. The epidemiology of this malignancy in the tongue and the floor of the mouth oscillate in the literature, but the high prevalence of these two locations in the literature is already well defined.

The racial distribution of the cohort did not show a significant difference between the two groups, with the majority of patients having brown skin, which is likely related to the great heterogeneity of the Brazilian population. In an epidemiological study of a population in Northeast Brazil published by Andrade et al., (2015), the brown/black skin color was the most frequently mentioned patient profile, also showing no significant association with an increased prevalence of oral SCC.

Regarding education level, patients with a history of smoking showed a significant association with a low education level. Although smoking generates a risk factor for oral SCC that is purely biological, it is known that the risk of developing oral cancer is increased in individuals with low education levels, low occupational classes and low incomes (Conway et al., 2008). Oral SCC is a type of cancer that has a relationship with social status, showing a worse prognosis in patients with lower socioeconomic status (Dantas et al., 2016).

Analysis of tumor staging and smoking history showed that in our sample, more smokers presented with cancer at a higher stage, which is in accordance with the literature, showing that these patients have a high frequency of advanced head and neck cancer (Dedivitis et al., 2004, Durazzo et al., 2005, Andrade et al., 2015). Therefore, smoking decreases the survival of patients with oral SCC, most likely because the tumor is at an advanced stage, making treatment efficacy and the possibility of cure quite difficult. Smoking history decreased the mean survival time by more than 30 months and the survival rate by more than 10% in our sample. Several factors besides smoking are necessary for the initiation, promotion and progression of cancer; however, systematic literature reviews have shown smoking as an important risk factor not only for the development but also for the worsening of the prognosis of oral cancer (Maynes et al., 2009).

Sex, age, race and tumor location did not significantly influence the survival of the patients in the present sample; however, age over 65 years old was an independent factor associated with a worse prognosis. Age-adjusted mortality rates were higher for men than for women (Cook et al., 2011), and aging may be a determinant of worse survival in patients with oral SCC, as noted in several studies (Kente et al., 2015; Van et al., 2015).

In relation to education level, illiterate patients have a lower mean survival time, and it is known that a low level of education is associated with reduced access to information about the disease in general, as well as delayed diagnosis and treatment (Conway et al., 2008; Wunsch-Filho et al., 2002). Therefore, some authors may consider oral cancer as a disease that is characteristic of people with low economic and educational levels (Madani et al., 2010), leading to diagnosis at more advanced stages and influencing the form of treatment and the patient prognosis. As observed in our cohort, smoking patients presented tumors at a higher stage and have a decreased survival, which is in line with the results of other studies (Razak et al., 2010; Wong et al., (2006).

Preliminary studies have demonstrated that smoking cessation promotes a significant reduction in the mortality rate, as well as produces a number of health benefits, which may be an important low-cost tool for improving the prognosis of SCC treatment in smokers (Sales et al., 2006). In our study, smoking showed an independent association with a low education level and a history of alcoholism, and survival was negatively associated with age and tumor size, which were more prevalent in smokers. Hence, we suggest that smoking history is an indirect determinant of worse survival, which can be used as a social prognostic marker.

In conclusion, the present study, despite its limitations of being a retrospective study of medical records, which are based on memory bias and response to inquiry (smoking history), has demonstrated that smoking history is an important driver of higher tumor stage that is associated with worse survival in patients with oral SCC.

## References

[B1] Al-Jaber A, Al-Nasser L, El-Metwally A (2016). Epidemiology of oral cancer in Arab countries. Saudi Med J.

[B2] Alvarenga LM, Ruiz MT, Bertelli ECP (2008). Epidemiologic evaluation of head and neck patients in a university hospital of Northwestern São Paulo State. Rev Bras Otorrinolaringol.

[B3] Andrade JO, Santos CA, Oliveira MC (2015). Associated factors with oral cancer: a study of case control in a population of the Brazil’s Northeast. Rev Bras Epidemiol.

[B4] Castagnola P, Gandolfo S, Malacarne D (2017). DNA aneuploidy relationship with patient age and tobacco smoke in OPMDs/OSCCs. PLoS One.

[B5] Chen KM, Guttenplan JB, Zhang SM (2013). Mechanisms of oral carcinogenesis induced by dibenzo[a, l]pyrene:anenvironmental pollutant and a tobacco smoke constituent. Int J Cancer.

[B6] Conway DI, Petticrew M, Marlborough H (2008). Socioeconomic inequalities and oral cancer risk: a systematic review and meta-analysis of case-control studies. Int J.

[B7] Cook MB, McGlynn KA, Devesa SS, Freedman ND, Anderson WF (2011). Sex disparities in cancer mortality and survival. Cancer Epidemiol Biomarkers Prev.

[B8] Dantas TS, Silva PGB, Sousa EF (2016). Influence of educational level, stage, and histological type on survival of oral cancer in a Brazilian population a retrospective study of 10 years observation. Medicine.

[B9] Dedivitis RA, França CM, Mafra ACB, Guimarães FT, Guimarães AV (2004). Clinic and epidemiologic characteristics in the with squamous cell carcinoma of the mouth and oropharynx. Rev Bras Otorrinolaringol.

[B10] Dobrossy L (2005). Epidemiology of head and neck cancer: magnitude of the problem. Cancer Metastasis Rev.

[B11] Doku D, Darteh EK, Kumi-Kyereme A (2013). Socioeconomic inequalities in cigarette smoking among men: evidence from the 2003 and 2008 Ghana demographic and health surveys. Arch Public Health.

[B12] Durazzo MD, Araujo CEN, Neto JSB (2005). Clinical and epidemiological features of oral cancer in a medical school teaching hospital from 1994 to 2002: increasing incidence in women, predominance of advanced local disease, and low incidence of neck metastases. Clinics.

[B13] Ferreira MAF, Gomes MN, Michels FAS, Dantas AA, Latorre MRDO (2012). Social inequality in morbidity and mortality from oral and oropharyngeal cancer in the city of. Cad Saúde Pública.

[B14] Ha PK, Califano JA (2004). The role of human papillomavirusin oral carcinogenesis. Crit Rev Oral Biol Med.

[B15] Hecht SS (2008). Progress and challenges in selected áreas of tobacco carcinogenesis. Chem Res Toxicol.

[B16] Kente EE, Ambs A, Mitchell SA (2014). Health-related quality of life in older adult survivors of selected cancers: data from the SEER-MHOS linkage. Cancer.

[B17] Madani AH, Dikshit M, Bhaduri D, Jahromi AS, Aghamolaei T (2010). Relationship between Selected Socio-demographic factors an cancer of oral cavity – a case control study. Cancer Inform.

[B18] Mayne ST, Cartmel B, Kirsh V, Goodwin WJ (2009). Alcohol and tobacco use pre- and post-diagnosis and survival in a cohort of patients with early stage cancers of the oral cavity, pharynx and Larynx. Cancer Epidemiol Biomarkers Prev.

[B19] Mendez M, Carrard VC, Haas AN (2012). A 10-year study of specimens submitted to oral pathology laboratory analysis: lesion occurrence and demographic features. Bras Oral Res.

[B20] Motgi AA, Chavan MS, Diwan NN (2014). Assessment of cytogenic damage in the form of micronuclei in oral epitelial cells in patients using smokeless and smoked form of tobacco and non-tobacco users and its relevan ce for oral cancer. J Cancer Res Ther.

[B21] Phillips DH, Venitt S (2012). DNA and protein adducts in human tissues resulting from exposure to tobacco smoke. Int J Cancer.

[B22] Razak AA, Saddki N, Naing NN, Abdullah N (2010). Oral cancer survival among Malay patients in Hospital Universiti Sains Malaysia, Kelantan. Asian Pacific J Cancer Prev.

[B23] Sales MP, Figueiredo MRF, Oliveira MI, Castro HN (2006). Outpatient smoking cessation program in the state of Ceará, Brazil: patient profiles and factors associated with treatment success. J Bras Pneumol.

[B24] Santos LCO, Batista OM, Cangussu MCT (2010). Characterization of oral cancer diagnostic delay in the state of Alagoas. Braz J Otorhinolaryngol.

[B25] Shiu MN, Chen TH (2004). Impact of betel quid, tobacco and alcohol on three-stage disease natural history of oral leukoplakia and cancer: implication for prevention of oral cancer. Eur J Cancer Prev.

[B26] Simard EP, Torre LA, Jemal A (2014). International trends in head and neck cancer incidence rates: differences by country, sex and anatomic site. Oral Oncol.

[B27] Sreeramareddy CT, Pradhan PM, Sin S (2014). Prevalence, distribution, and social determinants of tobacco use in 30 sub-Saharan African countries. BMC Med.

[B28] Stewart BW, Wild CP (2014). World Cancer Report. Lyon: IARC Press.

[B29] Streppel MT, Boshuizen HC, Ocké MC, Kok FJ, Kromhout D (2007). Mortality and life expectancy in relation to long-term Cigarette, Cigar and Pipe Smoking: The Zutphen Study. Tob Control.

[B30] Tandon P, Dadhich A, Saluja H, Bawane S, Sachdeva S (2017). The prevalence of squamous cell carcinoma in different sites of oral cavity at our Rural Health Care Centre in Loni, Maharashtra – a retrospective 10-year study. Contemp Oncol (Pozn).

[B31] Van NA, Buffart LM, Brug J, Leemans CR, Verdonck-de Leeuw IM (2015). The association between health related quality of life and survival in patients with head and neck cancer: a systematic review. Oral Oncol.

[B32] Vargas-Ferreira V, Nedel F, Etges A (2012). Etiologic factors associated with oral squamous cell carcinoma in non-smokers and non-alcoholic drinkers: a brief approach. Braz Dent J.

[B33] Warnakulasuriya S, Sutherland G, Scully C (2005). Tobacco, oral cancer, and treatment of dependence. Oral Oncol.

[B34] Wienholds E, Kloosterman WP, Miska E (2005). MicroRNA expression in zebrafish embryonic development. Science.

[B35] Wong YK, Tsai WC, Lin JC (2006). Socio-demographic factors in the prognosis of oral cancer patients. Oral Oncol.

[B36] Wunsch-Filho V (2002). The epidemiology of oral and pharynx cancer in Brazil. Oral Oncol.

